# Similar Repair Effects of Human Placenta, Bone Marrow Mesenchymal Stem Cells, and Their Exosomes for Damaged SVOG Ovarian Granulosa Cells

**DOI:** 10.1155/2020/8861557

**Published:** 2020-12-03

**Authors:** Shuwen Chen, Yanbin Wang, Liming Liao, Li Meng, Juanjuan Li, Cheng Shi, Hongjing Han, Xiaofeng Zheng, Huan Shen

**Affiliations:** ^1^Reproductive Medical Center, Peking University People's Hospital, Peking University, Beijing 100044, China; ^2^School of Life Sciences, Peking University, Beijing 100191, China; ^3^Incinta Fertility Center, Torrance, CA, USA

## Abstract

**Background:**

This study is aimed at investigating the repairing effect of mesenchymal stem cells and their exosomes from different sources on ovarian granulosa cells damaged by chemotherapy drugs—phosphoramide mustard (PM).

**Methods:**

In this study, we choose bone marrow mesenchymal stem cells (BMSCs) and human placental mesenchymal stem cells (HPMSCs) for research. Then, they were cocultured with human ovarian granulosa cells (SVOG) injured by phosphoramide mustard (PM), respectively. *β*-Galactosidase staining, flow cytometry, and Western blot were used to detect the changes in the senescence and apoptosis of SVOG cells before and after their coculture with the above two types of MSCs. Subsequently, exosomes from these two types of MSCs were extracted and added to the culture medium of SVOG cells after PM injury to test whether these two types of exosomes played a role similar to that of MSCs in repairing damaged SVOG cells.

**Results:**

PM treatment-induced apoptotic SVOG cells were significantly decreased after HPMSCs and BMSCs as compared with control group. After coculturing with these two types of MSCs, PM-treated SVOG cells showed significantly reduced senescence and apoptosis proportions as well as cleaved-Caspase 3 expression, and HPMSCs played a slightly stronger role than BMSCs in repairing SVOG cells in terms of the above three indicators. In addition, the ratios of senescent and apoptotic SVOG cells were also significantly reduced by the two types of exosomes, which played a role similar to that of MSCs in repairing cell damages.

**Conclusions:**

The results indicated that BMSCs, HPMSCs, and their exosomes all exerted a certain repair effect on SVOG cells damaged by PM, and consistent repair effect was observed between exosomes and MSCs. The repair effect of exosomes secreted from BMSCs and HPMSCs on the SVOG cells was studied for the first time, and the results fully demonstrated that exosomes are the key carriers for MSCs to play their role.

## 1. Introduction

The pool of primordial follicles formed in the ovary during the fetal period is the basis of female ovarian reserves. Starting from adolescence, only a few follicles survive and mature, and most of them will undergo degeneration and atresia at different stages of development [1–4]. The quality of ovarian reserves determines the reproductive lifespan [5, 6]. The follicle pool is typically depleted in a slow manner. However, certain external factors, especially chemotherapy drugs, may greatly accelerate the follicle pool depletion. The high gonadal toxicity of chemotherapy drugs can cause follicular failure, amenorrhea, infertility, and an eventual progression to premature ovarian failure (POF), leading to complete loss of fertility in women of childbearing age.

The growing cancer incidence across the globe with an increasingly younger onset age in recent years [7], combined with the massive use of chemotherapy and radiotherapy, has largely led to the ever-increasing POF cases, which has become a top focus of research [8]. However, the mechanism underlying the POF caused by chemotherapeutic drugs is still unclear [9]. There are two mainstream views: (1) POF is the result of follicular atresia caused by excessive senescence, apoptosis, and loss of granulosa cells (GCs) [10, 11]; and (2) POF is the result of excessive activation of dormant primordial follicles [12, 13]. In order to study the mechanism of chemotherapeutic drugs damaging ovarian function, this study chooses phosphoramide mustard (PM) to conduct in vitro cell experiments. PM as the in vitro metabolic activity product of cyclophosphamide is an important substance for cyclophosphamide to exert cytotoxicity, and it is more suitable for in vitro cell experiments than other chemotherapeutics. Current clinical treatment of POF mainly aims to improve the low estrogen symptoms in patients [14, 15], which cannot solve their infertility issues. Therefore, the treatment with mesenchymal stem cells (MSCs) that aims to restore ovarian fertility has attracted many researchers worldwide.

As a type of adult stem cells derived from mesoderm, MSCs are capable of self-renewal, multidirectional differentiation, and tissue reconstruction [16–19]. Past studies have confirmed that MSCs can play an effective role in treating POF [20–27]. However, many scholars are currently worried that the application of MSCs may entail risks such as tumorigenicity, posttransplantation infection, secondary injury, and progeny safety [28–30], which greatly limit the clinical application of MSCs. Exosomes are a type of substance carrier secreted extracellularly after the fusion of intracellular multilayer vesicles with cell membranes. Exosomes have a lipid bilayer structure and contain various cell-specific active substances such as nucleic acids, proteins, and lipids [31–34]. Exosomes are key participants in intercellular and even interorgan communication [35] and play an important role in numerous pathophysiological processes [36–42]. In the field of reproduction research, Nesrine et al. discovered in 2018 that the use of estrogen in conjunction with exosomes secreted by human umbilical cord MSCs can effectively reduce endometrial inflammation and fibrosis indicators (TNF-*α*, TNF-*β*, IL-1, IL-6, RUNX2, and collagen-1) and can regenerate damaged endometrium and reverse endometrial fibrosis [43]. However, at present, research on the application of exosomes in repairing germ cell damage and reconstructing reproductive functions is still in its early stage with little literature available and limited understanding regarding the role of exosomes. Therefore, it is important to explore the reproductive repair functions of exosomes derived from MSCs and search for a substance carrier with a variety of active factors and ovarian repair functions, thus paving the way for further study and application of safe cell-free therapy.

## 2. Materials and Methods

### 2.1. Culture and Characterization of BMSCs and HPMSCs

**Figure 1:**
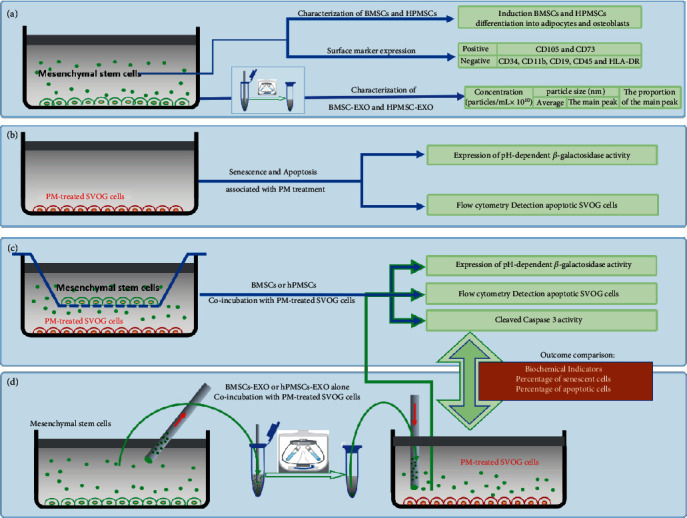
Schematic design of this experiment. (a) The characterization of BMSCs and HPMSCs. The separation and characterization of BMSC-EXO and HPMSC-EXO. (b) Senescence and apoptosis associated with PM-treated SVOG cells. (c) BMSCs or HPMSCs coincubation with PM-treated SVOG cells. (d) BMSCs-EXO or HPMSCs-EXO alone coincubation with PM-treated SVOG cells.

Bone marrow mesenchymal stem cells (BMSCs, Institute of Hematology, Peking University People's Hospital, Beijing, China) and human placental mesenchymal stem cells (HPMSCs, Boya Stem Cell Bank, Beijing, China) were placed in a complete medium of DMEM/F-12 (GIBCO, USA) containing 10% of fetal bovine serum (Hyclone, USA) and 1% penicillin-streptomycin (GIBCO, USA), before being transferred to a cell culture incubator. All cells were cultured at 37°C in a humidified atmosphere containing 5% CO2. The medium was changed once every two days (2 d). The cell growth status was observed under a microscope. When the confluence density of the cells reached 80–90%, they were passaged using routine subculture techniques, and some cells were cryopreserved at the same time. The cells were passaged continuously, and subsequent experiments were performed using P6–P9 generations of the cells. The content of MSC characterization included induction of adipogenic differentiation, induction of osteogenic differentiation, and the presence of surface markers of MSCs including CD105, CD73, CD34, CD11b, CD19, CD45, and HLA-DR (Abcam, USA) (Figure 1(a)).

### 2.2. Separation and Extraction of BMSC-EXO and HPMSC-EXO

BMSCs and HPMSCs (P6–P9 generations) were cultured at 37°C in a humidified atmosphere containing 5% CO2. When the cells reached 80–90% confluence, they were washed three times with PBS (Solarbio, Beijing, China) and then further cultured for 48 h in an exosome-free (EXO-free) serum-containing medium. Subsequently, the supernatant of the cell culture medium was collected, and the exosomes were extracted by ultrahigh speed gradient centrifugation. The specific steps for exosome extraction are as follows: first, an ultrahigh speed centrifuge was used to centrifuge the collected supernatant at 10,000 × g and 4°C for 30 min, and the supernatant was retained after centrifugation and filtered through a 0.22 *μ*m filter membrane to remove residual cells and cell debris. Second, the filtered supernatant was centrifuged again at 120,000 × g and 4°C for 1.5 h, and the supernatant was discarded after centrifugation while the precipitate was collected and resuspended in 15 mL of PBS by blowing. Finally, the suspension was centrifuged at 100,000 × g and 4°C for 1 h, and the resulting precipitates were exosomes. The exosomes were aliquoted and cryopreserved for future downstream experiments.

### 2.3. Characterization of BMSC-EXO and HPMSC-EXO

The characterization methods of exosomes included: assay of protein concentration using the BCA method; observation of the morphological characteristics of exosomes using transmission electron microscopy (TEM); assay of the expression of exosomal marker proteins CD81, TSG101, ALIX, and Calreticulin using Western blot; and assay of exosome diameter using nanoparticle tracking analysis (NTA).

### 2.4. Assay of BCA Protein Concentration

A standard curve was plotted according to the BCA assay kit (Solarbio, Beijing, China) to calculate the concentrations of BMSC-EXO and HPMSC-EXO.

### 2.5. Observation of Morphological Characteristics of Exosomes by TEM

A small amount of BMSC-EXO and HPMSC-EXO suspensions was pipetted onto the surface of a copper grid with an ultrathin carbon support film. After 1 min of adsorption, the excess suspension on the edge of the copper grid was removed with a piece of clean filter paper, and the copper grid was washed by immersing in the filtered PBS for 5–10 s; then, the copper grid was negatively stained three times with a 2% uranium acetate staining solution. After the excess staining solution on the edge of the copper grid was removed, the copper grid was left at room temperature and dried in the horizontal position. A 200 kV transmission electron microscope was used to observe and photograph the copper grid.

### 2.6. Assay of the Expression of Exosome-Specific Proteins Using Western Blot

BMSC-EXO, HPMSC-EXO, and cellular proteins in the control group were collected. The cellular proteins in the control group were obtained by using a conventional method and used as a positive control of exosomes. 30 *μ*g of the test sample was pipetted into a 12% SDS-PAGE gel (Solarbio, Beijing, China) by a pipette, and the electrophoresis was carried out at constant voltages of 80 V and 120 V successively for 0.5 h and 1 h, respectively, using an electrophoresis instrument; then, the samples were electro-transferred onto PVDF membranes at a constant current of 200 mA for 1.5 h (the time of electrophoresis and transfer was adjusted appropriately according to the molecular weight of the proteins); in the next step, the PVDF membranes were placed into a blocking solution containing 5% skimmed milk powder for 1 h of blocking at room temperature; after the PVDF membranes were washed using a 1 × TBST buffer (Solarbio, Beijing, China), antibodies against CD81, TSG101, ALIX, and Calreticulin (Proteintech, USA) were added, and the PVDF membranes were incubated overnight at 4°C on a shaker; after the PVDF membranes were washed using the 1 × TBST buffer, they were incubated with horseradish peroxidase-labeled anti-IgG antibodies for 1 h at room temperature under gentle shaking. Finally, after the PVDF membranes were washed using the 1 × TBST buffer, an ECL chemiluminescence reagent was added for color development, and the absorbance of each band was measured for quantitative analysis.

### 2.7. Assay of Exosome Diameter Using NTA

A laser light source was used to illuminate nanoparticle suspension, and the light scattered by the nanoparticles was detected to count the number of scattering nanoparticles and calculate the nanoparticle concentration. In this way, a particle matrix Zeta View PMX 110 was used to determine the concentration of exosomes obtained by separation at an emission wavelength of 405 nm, and the exosomes were diluted with PBS to 1 × 10^7^–1 × 10^9^ particles/mL. In addition, the size and mass of the exosomes were measured. At the same time, the trajectory of exosomal movement was analyzed.

### 2.8. Treatment of Ovarian Granulosa-Lutein (SVOG) Cells Using PM

SVOG cells (Shenzhen Huatuo Biotechnology Co., Ltd., Shenzhen, China) were seeded at a density of 1 × 10^5^ cells per well in six-well plates and were cultured in a complete medium of DMEM (GIBCO, USA) containing 10% of fetal bovine serum (Hyclone, USA) and 1% penicillin-streptomycin (GIBCO, USA). All cells were cultured at 37°C in a humidified atmosphere containing 5% CO2. Following incubation, PM (Shenzhen Lijing Biochemical Technology Co., Ltd., Shenzhen, China) was added to the medium to a final concentration of 30 *μ*mol/L, and the cells were cultured for 24 h for subsequent experiments (Figure 1(b)).

### 2.9. Coculture of PM-Treated SVOG Cells with MSCs and Exosomes

Transwell culture plates (0.4 *μ*m) were used in this experiment. The semipermeable membrane of each Transwell could separate the upper and lower layers of the cells so that the cells could not migrate between the upper and lower chambers of the Transwell at random. Nevertheless, the cytokines secreted by the cells in the upper and lower chambers of the Transwell could pass through the semipermeable membrane, thus achieving the coculture of SVOG cells with BMSCs and HPMSCs. The experiment was divided into 6 groups: (1) untreated group of SVOG cells (blank control group), (2) group of SVOG cells treated with PM, (3) coculture group of PM − treated SVOG cells + BMSCs, (4) coculture group of PM − treated SVOG cells + HPMSCs, (5) coculture group of PM − treated SVOG cells + BMSC‐EXO, and (6) coculture group of PM − treated SVOG cells + HPMSC‐EXO. Three parallel wells were set to each group, and the subsequent experiments were performed after the cells in each group were further cultured for 48 h in a DMEM/F-12 complete medium (Figures 1(c) and 1(d)).

### 2.10. Detection of Cell Senescence by *β*-Galactosidase Staining

The cell culture medium in the 6-well culture plates was aspirated, and the cells were washed once with PBS, before 1 mL of *β*-galactosidase staining and fixation solution (Beyotime, Shanghai, China) was added to each well to fix the cells at room temperature for 15 min. The cell fixation solution was aspirated, and the cells were washed 3 times with PBS for 3 min each time. The PBS in the wells was aspirated before 1 mL of staining working solution was added to each well. The cells were incubated in a 37°C incubator (with no carbon dioxide) overnight, and the 6-well plates were sealed with parafilm to prevent evaporation. The amount of dark blue particles generated in the culture plates was observed under an ordinary light microscope, and images were taken for analysis.

### 2.11. Detection of Apoptosis by Flow Cytometry

Cells were prepared using a FITC Annexin V assay kit (BD, USA) as recommended by the manufacturer. The cells were washed twice with cold PBS and then resuspended in a 1× binding buffer to a concentration of 1 × 10^6^ cells/mL; 100 *μ*L of the cell suspension (containing 1 × 10^5^ cells) was transferred to a 5 mL culture tube, and 5 *μ*L of FITC Annexin V and 5 *μ*L of PI were added to the tube. After gentle rotation of the cells in the tube, the cells were incubated for 15 min at room temperature (25°C) in a dark environment; then, 400 *μ*L of the 1× binding buffer was added to each tube, and the cells were loaded onto a flow cytometer within 1 h for detection. The source data were analyzed using the FlowJo10.0.7 software.

### 2.12. Detection of Protein Expression of Apoptosis-Associated Caspase 3 by Western Blot

For cell protein extraction from each group, after the cell culture medium was discarded, the cells were washed 2–3 times with prechilled PBS, and 150 *μ*L/well of a RIPA lysis buffer was added to the 6-well plates and mixed well. The cells were placed on a shaker on ice and lysed for 30 min at 150 × g, followed by 30 min of centrifugation at 12,000 × g and 4°C, and the supernatant was the fraction of cellular proteins. The protein concentration was determined by the BCA method for quantitative analysis, and the protein samples were stored at -80°C for future use. 30 *μ*g of each protein sample was added into a 12% SDS-PAGE gel, and the electrophoresis was carried out at constant voltages of 80 V and 120 V successively for 0.5 h and 1 h, respectively; then, the samples were electro-transferred onto PVDF membranes at a constant current of 200 mA for 1.5 h (the time of electrophoresis and transfer was adjusted appropriately according to the molecular weight of the proteins); in the next step, the PVDF membranes were placed into a blocking solution containing 5% skimmed milk powder for 1 h of blocking at room after the PVDF membranes were washed using a 1 × TBST buffer, antibodies against Caspase 3 and *β*-tublin (Proteintech, USA) were added, and the PVDF membranes were incubated overnight at 4°C on a shaker; after the PVDF membranes were washed using the 1 × TBST buffer, they were incubated with horseradish peroxidase-labeled anti-IgG antibodies for 1 h at room temperature under gentle shaking. Finally, after the PVDF membranes were washed using the 1 × TBST buffer, an ECL chemiluminescence reagent was added for color development, and the absorbance of each band was measured for quantitative analysis.

### 2.13. Statistical Analysis

All statistical analyses were performed with the SPSS software. The data are expressed as the mean ± SD of at least three independent experiments *in vitro*. Comparisons were performed using a two-tailed *t*-test or one-way ANOVA for experiments with more than two subgroups. The error bars indicate the standard deviation from the mean of triplicate measurements. Asterisks indicate significant differences (∗*P* < 0.05; ∗∗*P* < 0.01; ∗∗∗*P* < 0.001) compared with the corresponding control.

## 3. Results

### 3.1. Culture and Characterization of BMSCs and HPMSCs

**Figure 2:**
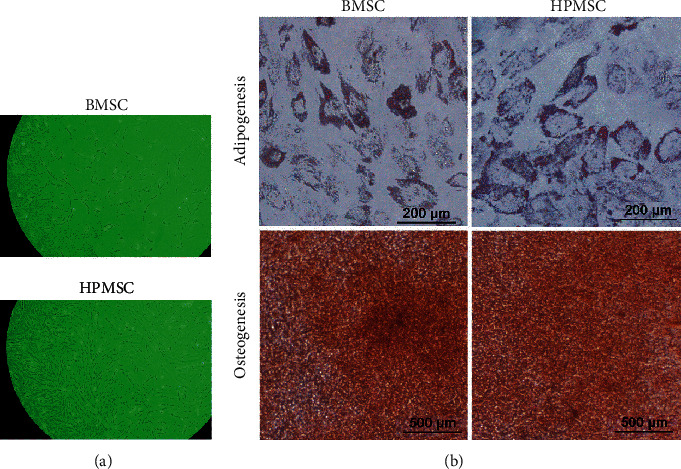
(a) The morphological characteristics of two types of MSCs observed under a microscope (100x): a fibroblast-like long-spindle shape, adherent and vortex-shaped growth, and orderly arrangement. (b) Staining for the induction of adipogenic and osteogenic differentiation of two types of MSCs.

**Figure 3:**
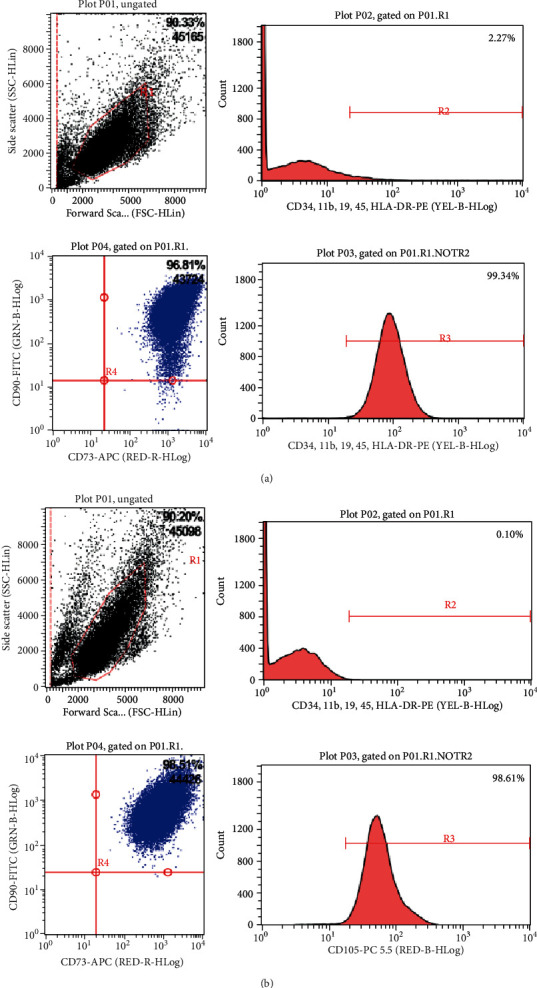
The surface markers of two types of MSCs characterized by flow cytometry: strong expression of CD105 and CD73 and no expression of CD34, CD11b, CD19, CD45, and HLA-DR. (a) BMSC; (b) HPMSC.

The P6 generation of the two types of MSCs was placed under a 100x optical microscope and photographed using the software. It was found that the two types of MSCs—BMSCs and HPMSCs—showed uniform morphology and good adherent growth, and both cells showed a fibroblast-like long spindle-shape with vortex-like growth and orderly arrangement (Figure 2(a)). 14 d after induction of adipogenic differentiation of the cells, the oil red O staining showed the formation of standard lipid droplets; 14 d after the induction of osteogenic differentiation, the alizarin red staining showed dark red nodules (Figure 2(b)). The surface markers of two types of MSCs were characterized by flow cytometry. The results indicated that the surface of these cells showed strong expression of CD105 and CD73, with undetectable the expression of CD34, CD11b, CD19, CD45, and HLA-DR (Figure 3).

### 3.2. Extraction and Characterization of BMSC-EXO and HPMSC-EXO

**Figure 4:**
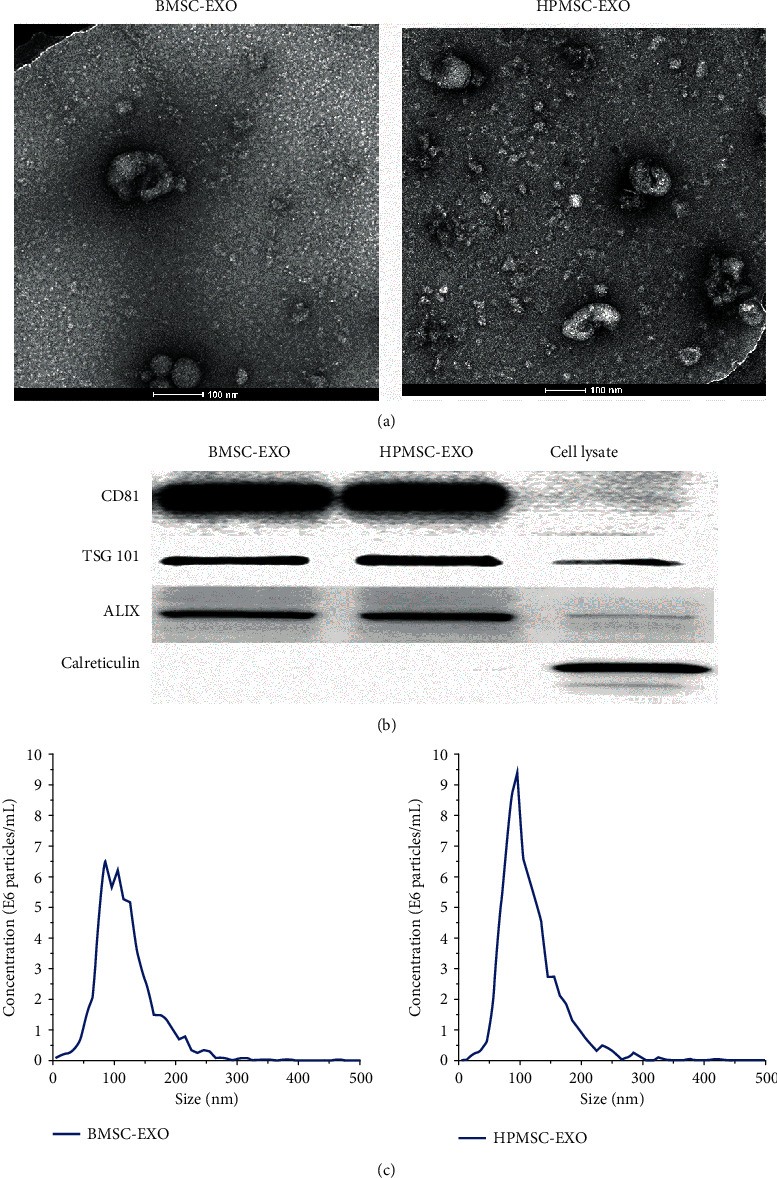
(a) Morphological characteristics of exosomes observed by TEM. (b) Marker protein expression of exosomes secreted from two types of MSCs detected by Western blot. (c) Exosomes characterized by NTA.

The exosomes of MSCs were extracted by ultrahigh speed gradient centrifugation and observed by TEM. The results showed that the exosomes of two types of MSCs were different in size, with a round or oval shape and a diameter of about 100 nm. Showing an obvious double-layer vesicular membrane structure, the exosomes were distributed in single form or in clusters, with a clear background and only a few contaminants (Figure 4(a)). The exosome-specific marker proteins were detected by Western blot. The results showed that both types of exosomes had strong expression of positive protein markers CD81, TSG101, and ALIX, and there was no expression of the negative protein marker Calreticulin (Figure 4(b)). The diameters of exosomes were assayed by NTA. The concentration of exosomes secreted by the BMSCs was 2.8 × 10^10^ (particles/mL), indicating that a large number of particles were extracted. The average particle size of these exosomes was 118.8 nm, the main peak of particle size was 99.7 nm, and the proportion of the main peak was 98.2%, indicating that the particle sizes of these exosomes were consistent with the theoretical size of exosomes and the sizes were distributed in a narrow range. The concentration of exosomes secreted by the HPMSCs was 3.4 × 10^11^ (particles/mL), indicating that a large number of particles were extracted. The average particle size of these exosomes was 115.9 nm, the main peak of particle size was 94.5 nm, and the proportion of the main peak was 94.9%, indicating that the particle sizes of these exosomes were consistent with the theoretical size of exosomes and the sizes were distributed in a narrow range (Figure 4(c), Table 1).

### 3.3. Changes in Senescence and Apoptosis of PM-Treated SVOG Cells

**Figure 5:**
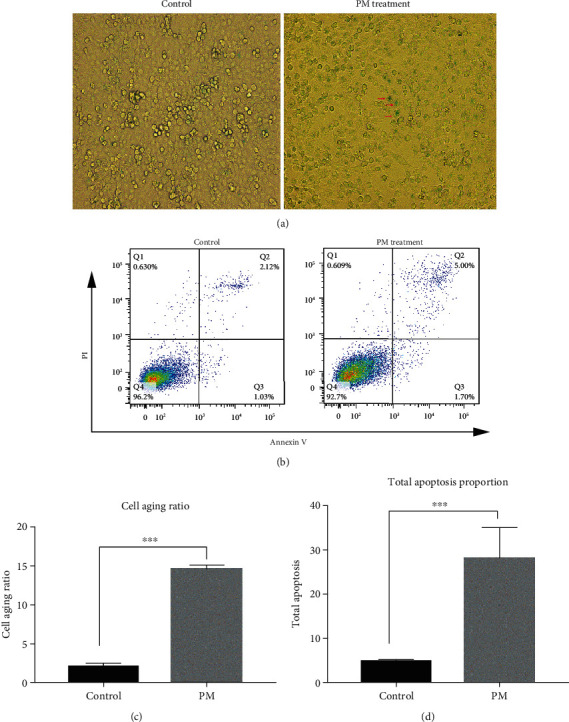
(a, c) The number of dark blue particles in the PM-injured SVOG cells significantly increased (shown by the red arrows), the proportion of senescent cells in the PM treatment group significantly increased, and the difference was statistically significant. ^∗∗∗^*P* < 0.001. (b, d) The proportion of apoptotic SVOG cells (the total apoptosis calculated as (Q2 + Q3)) significantly increased after PM injury, and the difference was statistically significant. ^∗∗∗^*P* < 0.001.

After SVOG cells were treated with PM, *β*-galactosidase staining was used to detect the changes in the senescence of the cells before and after the treatment. The results showed that the number of dark blue particles in the PM-treated SVOG cells increased significantly. The semiquantitative analysis showed that the proportion of senescent cells in the PM treatment group significantly increased, and the difference was statistically significant (*P* < 0.001) (Figures 5(a) and 5(c)).

After SVOG cells were treated with PM, flow cytometry was used to detect the changes in the apoptosis of the cells. The results showed that the proportion of apoptotic SVOG cells significantly increased after the PM treatment, and the difference was statistically significant (*P* < 0.001) (Figures 5(b) and 5(d)).

### 3.4. Effects of BMSCs and HPMSCs on Senescence and Apoptosis of PM-Treated SVOG Cells

**Figure 6:**
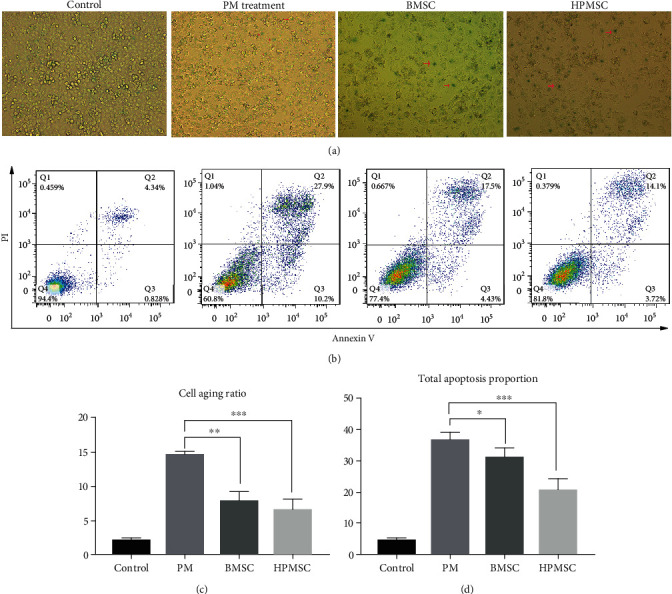
(a, c) The changes to the senescence of PM-treated SVOG cells after coculturing with BMSCs and HPMSCs (red arrows indicate typical senescent cells). The differences were statistically significant. ^∗∗^*P* < 0.01; ^∗∗∗^*P* < 0.001. (b, d) The changes to the apoptosis of PM-treated SVOG cells after coculturing with BMSCs and HPMSCs. The calculation formula of the total proportion of apoptosis was (the total apoptosis calculated as (Q2 + Q3)). The differences were statistically significant. ^∗^*P* < 0.05; ^∗∗∗^*P* < 0.001.

After PM-treated SVOG cells were cocultured with BMSCs and HPMSCs, *β*-galactosidase staining was used to detect the number of dark blue particles in the SVOG cells to show the changes in the senescence of these cells. The results showed that the proportion of cell senescence in the BMSC group significantly decreased compared with that in the PM treatment group, and the difference was statistically significant (*P* < 0.01); similarly, the proportion of cell senescence in the HPMSC group significantly decreased compared with that in the PM treatment group, and the difference was statistically significant (*P* < 0.001) (Figures 6(a) and 6(c)). In addition, the effect of senescence repair in the HPMSC group was slightly stronger than that in the BMSC group, but the difference was not statistically significant (*P* > 0.05).

The changes in the apoptosis of SVOG cells were detected using flow cytometry. The results showed that the proportion of cell apoptosis in the BMSC coculture group significantly decreased compared with that in the PM treatment group, and the difference was statistically significant (*P* < 0.05). Similarly, the proportion of cell apoptosis in the HPMSC coculture group significantly decreased compared with that in the PM treatment group, and the difference was statistically significant (*P* < 0.001) (Figures 6(b) and 6(d)). In addition, the effect of apoptosis repair in the HPMSC group was slightly stronger than that in the BMSC group, but the difference was not statistically significant (*P* > 0.05).

**Figure 7:**
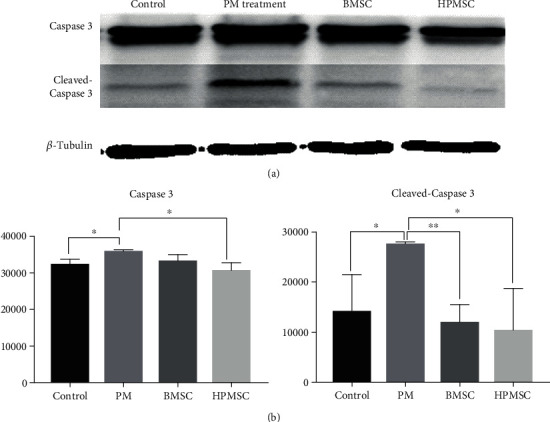
(a) Detection of differences in the protein expression of apoptosis-associated Caspase 3 zymogen and cleaved-Caspase 3 in 4 groups of cells by Western blot. (b) Analysis of difference in gray values. The differences were statistically significant. ^∗^*P* < 0.05; ^∗∗^*P* < 0.01.

Western blot was used to detect the difference in the expression of the apoptosis-associated protein Caspase 3. The results showed that the expression of cleaved-Caspase 3 in SVOG cells in the BMSC coculture group significantly decreased compared with that in the PM group, indicating that the apoptotic SVOG cells decreased and the functions of SVOG cells were restored. The difference in the gray value analysis of the two groups was statistically significant (*P* < 0.01). The expression of Caspase 3 zymogen and cleaved-Caspase 3 in the SVOG cells in the HPMSC coculture group significantly decreased compared with that in the PM group, indicating that the apoptotic SVOG cells decreased and the functions of SVOG cells were restored. The difference in the gray value analysis of the two groups was statistically significant (*P* < 0.05) (Figures 7(a) and 7(b)). The results of Western blot could be corroborated with the above results of flow cytometry.

### 3.5. Effects of BMSC-EXO and HPMSC-EXO on the Senescence and Apoptosis of SVOG Cells after PM Injury

**Figure 8:**
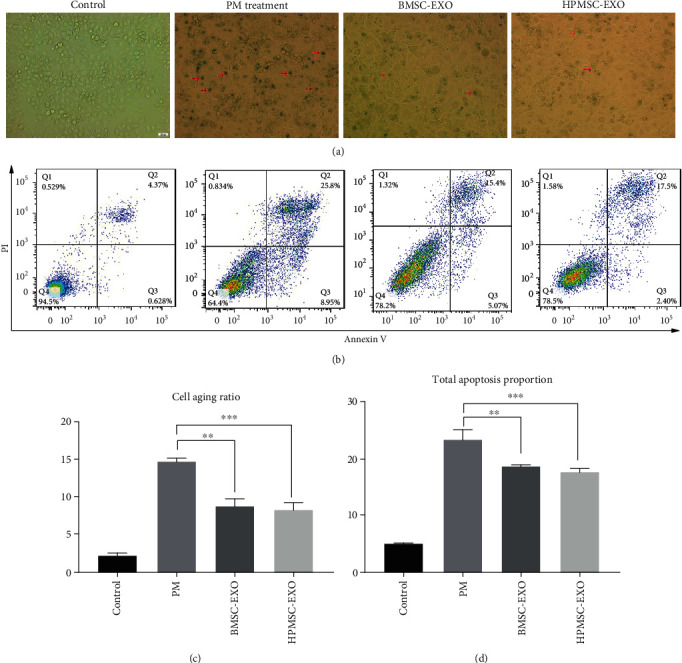
(a, c) The changes to the senescence of PM-treated SVOG cells after the addition of BMSC-EXO and HPMSC-EXO (red arrows indicate typical senescent cells). The differences were statistically significant. ^∗∗^*P* < 0.01; ^∗∗∗^*P* < 0.001. (b, d) The changes to the apoptosis of PM-treated SVOG cells after the addition of BMSC-EXO and HPMSC-EXO. The calculation formula of the total proportion of apoptosis was (the total apoptosis calculated as (Q2 + Q3)). The differences were statistically significant. ^∗^*P* < 0.01; ^∗∗∗^*P* < 0.001.

BMSC-EXO and HPMSC-EXO were added to the culture medium of PM-treated SVOG cells, and a *β*-galactosidase staining was used to detect the number of dark blue particles in SVOG cells to show the changes in cell senescence. The results showed that the proportion of cell senescence in the BMSC-EXO group significantly decreased compared with that in the PM treatment group, and the difference was statistically significant (*P* < 0.01); similarly, the proportion of cell senescence in the HPMSC-EXO group significantly decreased compared with that in the PM treatment group, and the difference was statistically significant (*P* < 0.001) (Figures 8(a) and 8(c)). In addition, the effect of senescence repair in the HPMSC-EXO group was slightly stronger than that in the BMSC-EXO group, but the difference was not statistically significant (*P* > 0.05).

The changes in the apoptosis of SVOG cells were detected using flow cytometry. The results showed that the proportion of cell apoptosis in the BMSC-EXO group significantly decreased compared with that in the PM treatment group, and the difference was statistically significant (*P* < 0.05). Similarly, the proportion of cell apoptosis in the HPMSC-EXO group significantly decreased compared with that in the PM treatment group, and the difference was statistically significant (*P* < 0.01) (Figures 8(b) and 8(d)). In addition, the effect of apoptosis repair in the HPMSC-EXO group was slightly stronger than that in the BMSC-EXO group, but the difference was not statistically significant (*P* > 0.05).

**Figure 9:**
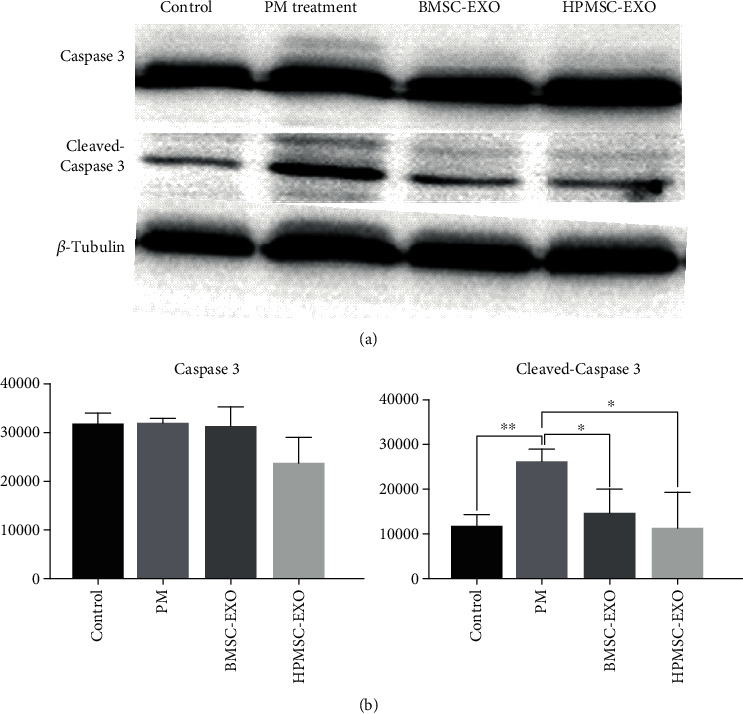
(a) Detection of differences in the protein expression of apoptosis-associated Caspase 3 zymogen and cleaved-Caspase 3 in 4 groups of cells by Western blot. (b) Analysis of difference in gray values. The differences were statistically significant. ^∗^*P* < 0.05; ^∗∗∗^*P* < 0.001.

Western blot was used to detect the difference in the expression of the apoptosis-associated protein Caspase 3. The results showed that the expression of cleaved-Caspase 3 in SVOG cells in the BMSC-EXO group significantly decreased compared with that in the PM group, indicating that the apoptotic SVOG cells decreased and the functions of SVOG cells were restored. The difference in the gray value analysis of the two groups was statistically significant (*P* < 0.05). The expression of cleaved-Caspase 3 in the SVOG cells in the HPMSC-EXO group significantly decreased compared with that in the PM group, indicating that the apoptotic SVOG cells decreased and the functions of SVOG cells were restored. The difference in the gray value analysis of the two groups was statistically significant (*P* < 0.05) (Figures 9(a) and 9(b)). The results of Western blot could be corroborated with the above results of flow cytometry.

### 3.6. Comparison of the Effects of Two Types of MSCs and Their Secreted Exosomes

**Figure 10:**
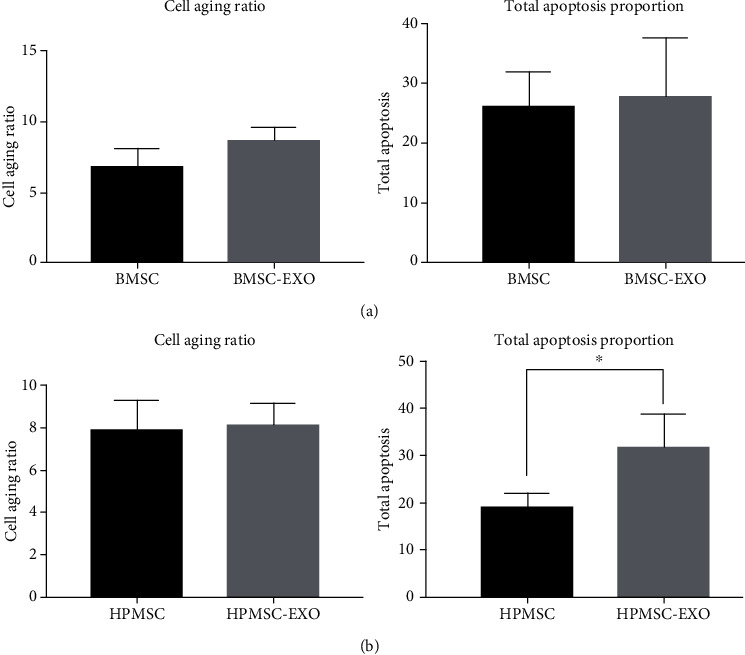
(a) Comparison of the effects of BMSCs and their secreted exosomes. (b) Comparison of the effects of HPMSCs and their secreted exosomes.

The effects of BMSCs, HPMSCs, and their exosomes on the senescence and apoptosis of PM-treated SVOG cells were compared. The results showed consistent repair effects of BMSCs, HPMSCs, and their exosomes on cell senescence, and their differences were not statistically significant (*P* > 0.05). The comparison of the proportion of apoptotic cells showed that HPMSCs were associated with better repair effects than their exosomes, and the difference was statistically significant (*P* < 0.05); BMSCs and their secreted exosomes had consistent repair effects on apoptosis, and their differences were not statistically significant (*P* > 0.05) (Figures 10(a) and 10(b)).

## 4. Discussion

Granular cells (GCs) play important roles in a series of processes such as the activation, growth and development, and atresia of follicles. The proliferation and differentiation of GCs are an important part of follicular development. As the pregranular cells change from a flat shape to a cubic shape, the primordial follicles are activated from their dormant state [44]. Subsequently, the activated primordial follicles become growing follicles. However, most of the growing follicles will undergo atresia, and only very few primordial follicles can become dominant follicles and continue to grow and mature until ovulation [45]. At the same time, GCs can secrete a variety of factors, such as inhibins and activins, to jointly regulate follicular development [46]. Many studies have confirmed that the selection and atresia of follicles are determined by the apoptosis of GCs [47, 48]. In addition, one of the mainstream views on the mechanism underlying the onset of POF caused by chemotherapeutic drugs is that POF is the result of follicular atresia caused by the excessive senescence, apoptosis, and loss of GCs [10, 11]. Therefore, it is very important to improve the ovarian functions and restore the fertility of patients by inhibiting the apoptosis of GCs. Therefore, in this study, an ovarian GC line SVOG was used as the model cell for in vitro experiments. By observing the changes in the senescence and apoptosis of GCs after injury and repair, a possible mechanism underlying the use of MSCs to treat POF was explored.

Apoptosis, also called programmed cell death, is an important cellular process involved in the homeostasis of multicellular organisms. The strict regulation of apoptosis has been involved in many human diseases [49]. Apoptosis is a dynamic process that involves the regulation of the expression of a series of genes, signal transduction, and a cascade of reactions involving multiple enzymes. Apoptotic signaling pathways include the death receptor pathway, mitochondrial pathway, and endoplasmic reticulum pathway, all of which eventually converge in the caspase cascade. As the executors of the apoptotic signaling pathways, cysteine-aspartic proteases [50] (caspases) are usually present in the form of zymogens and can mediate the protease cascade reactions after being activated. Among various caspases, Caspase 3 is a key effector molecule of apoptosis and plays a role in all of the death receptor pathway, mitochondrial pathway, and endoplasmic reticulum pathway. In the early stage of apoptosis, Caspase 3 (32 kD) is activated and cleaved by its upstream signal molecules to form a large 17 kD subunit and a small 12 kD subunit. Therefore, cleaved-Caspase 3 can be used as a marker of apoptosis.

MSCs have attracted widespread attention due to their potential of self-renewal and multidirectional differentiation, as well as good application prospects in the treatment of POF. The therapeutic effects of MSCs from various sources, including BMSCs [23] and HPMSCs [24, 25], on the treatment of POF have been validated. BMSCs are the earliest and most frequently studied MSCs. First discovered in rat bone marrow by Friedenstein and other researchers in 1966, BMSCs were characterized as fibroblast-like cells with cloning potential [51]. BMSCs have the characteristics of low immunogenicity, multidirectional differentiation, and homing [52], and their migration and high proliferation abilities ensure that the cells can reach the injured tissue to play a protective or repairing role. In addition, BMSCs can also secrete various paracrine cytokines, with the potential to repair the ovarian structure and improve ovarian endocrine functions [53]. However, the use of BMSCs is limited by the invasiveness in clinical applications. HPMSCs are also pluripotent nonhematopoietic progenitor cells. Separated from placental tissues that are usually discarded after delivery, HPMSCs have high differentiation and proliferation potential. HPMSCs are capable of self-renewal and differentiation into mesenchymal lineages, with good immunomodulatory effects [54], as well as the characteristics of easy separation, low viral infection rates, and low immunogenicity [55]. However, due to the heterologous source of HPMSCs, there are concerns about safety, immunogenicity, and integration of heterologous genes.

At present, the mechanism by which MSCs play a role in tissue repair has not been fully elucidated, and there is no evidence indicating that transplanted MSCs can directly differentiate into GCs or oocytes to replace damaged cells with necessary functions. Some researchers have suggested that in the repair of ovarian damage caused by chemotherapeutic drugs, MSCs play their role by secreting factors conducive to cell survival, such as growth factors VEGF, IGF-1, and HGF [56], which can improve the ovarian microenvironment, thereby effectively restoring ovarian functions. As important carriers of intercellular communication, exosomes may be the key substances for MSCs to play their role.

As a type of membranous vesicles that can be actively secreted by almost all types of eukaryotic cells, exosomes are uniform in size with a regular round shape and a diameter of 30–150 nm. Exosomes are a type of extracellular vesicles (EVs) [32]. EVs have been validated to play a role in fertilization and mating behaviors [57]. In early embryos, EVs released by embryonic stem cells in the inner cell mass can promote the migration and implantation of embryos [58]. EVs can also affect the mating behavior [59], as well as tail chasing behavior and tracking behavior among males [60]. At present, exosomes are widely used, with good progress made in life science and medical studies. Studies have shown that, by releasing exosomes containing proteins and RNAs, tumors can change the microenvironment before tumor metastasis to assist tumor metastasis [61]. In addition, Belov et al. have confirmed that as a new type of liquid biopsy markers, exosomes are gradually replacing invasive diagnostic methods such as tissue biopsy [62]. The researchers fixed antibodies against known tumor surface markers on a chip and compared tumor cells with exosomes secreted by these cells. They found that about 40% of the surface markers of cancer cells were present in their exosomes, indicating that the detection of exosome surface markers could be used to replace tissue biopsy of tumor cells to a certain extent. Exosomes can also play the role of carriers in tumor treatment. Yim et al. used a blue light to control the precise delivery of protein drugs by exosomes to achieve targeted treatment of tumor cells [63]. At the same time, the research team of Qian found that the exosomes secreted from MSCs could transfer host molecules among different cells to inhibit virus replication, thereby achieving antiviral effects [64]. Researchers at the University of Ghent in Belgium have found [65] that the exosomes released by choroid plexus epithelial cells were a new mechanism of blood-brain communication, and the exosomes released from choroid plexus could enter the brain parenchyma and be uptaken by astrocytes and microglial cells to suppress miRNA target genes and upregulate inflammatory genes, thus causing systemic inflammatory diseases. Besides, the blockage of exosome secretion can reduce brain inflammation. This study opened up a new approach for the treatment of systemic inflammatory diseases.

The results of this study have showed that the exosomes secreted by BMSCs and HPMSCs also exerted a certain repair effect on PM-treated SVOG cells, and the BMSCs and exosomes secreted by such BMSCs showed identical abilities in reducing cell senescence and apoptosis. However, the antiapoptosis ability of the exosomes secreted by HPMSCs was weaker than that of the HPMSCs themselves, but the antisenescence ability of the exosomes secreted by HPMSCs was identical with that of such HPMSCs. The results of this study have fully demonstrated that exosomes are the key carriers for MSCs to play their role. The use of exosomes to treat GCs damaged by chemotherapeutic drugs can basically achieve the same effect achieved by the use of MSCs themselves. Using exosomes can successfully avoid many risks of the MSC therapy, thus providing a theoretical basis for using exosomes as a new type of “cell-free” therapy to replace the MSC therapy. The involvement of exosomes represents a fundamental change in our current understanding of the paracrine effect of MSCs on tissue and cell repair. This is because the current mainstream research on paracrine mechanisms is still limited to the extracellular signaling mechanisms mediated by cytokines, chemokines, or growth factors. This study highlights the new role of exosomes as mediators in tissue repair, providing a novel perspective on intercellular mediation of tissue damage repair, as well as a new approach for the development of biologics for tissue repair. As lipid vesicles, exosomes are ideal carriers for intercellular communication. By rapidly transferring functional proteins in cells, exosomes can physiologically respond to repair and complete the repair in real time. Recent studies have shown that, in addition to proteins, exosomes can also act as secretory factors to affect the subtype of receptor cells by transferring their internal RNAs, especially miRNAs [66], or by binding to the surface receptors of specific cells to change their protein and gene expression so as to participate in signal transduction to regulate target cells and play an important role in tissue repair. If the above hypothesis is further confirmed by future research, it will provide a new approach for the development of biological agents in the future. Nevertheless, this study still has certain limitations. The most obvious one may be the lack of in vivo studies. The efficacy and safety of MSCs and their secreted exosomes in the treatment of animal models with chemotherapy-induced POF should be determined. In addition, at present, the extraction of sufficient exosomes from the MSC culture medium requires considerable human and financial resources. Therefore, these limitations should be explored so that follow-up studies will be optimized.

## 5. Conclusion

As shown in the study results, BMSCs, HPMSCs, and their exosomes can significantly reduce the proportions of senescent and apoptotic SVOG cells after PM injury, showing a certain repair effect. The repair effects of the exosomes and their MSCs are consistent. This work is the first comparative study of the repairing effect of exosomes secreted by BMSC and HPMSC on SVOG cells and shows that exosomes may be the key carrier for MSC to function. At the same time, our research on mesenchymal stem cell exosomes can provide new ideas for the future development of new biological agents.

## Figures and Tables

**Table 1:** Exosomes characterized by NTA.

	Concentration (particles/mL × 10^10^)	Particle size (nm)		The proportion of the main peak
		Average	The main peak	
BMSCs	2.8	118.8	99.7	98.2%
HPMSCs	34	115.9	94.5	94.9%,

## Data Availability

Data and materials will be made available upon request via email to first author (chenshuwen@bjmu.edu.cn) and corresponding author (rmivf@sina.com).

## Abbreviations

MSCs: Mesenchymal stem cells
BMSCs: Bone marrow mesenchymal stem cells
HPMSCs: Human placental mesenchymal stem cells
PM: Phosphoramide mustard
POF: Premature ovarian failure
EXO-free: Exosome-free
NTA: Nanoparticle tracking analysis
TEM: Transmission electron microscopy.

## References

[1] Janas P., Kucybała I., Radoń-Pokracka M., Huras H. (2018). Telocytes in the female reproductive system: an overview of up-to-date knowledge. *Advances in Clinical and Experimental Medicine*.

[2] Ozturk S. (2019). The translational functions of embryonic poly(A)-binding protein during gametogenesis and early embryo development. *Molecular Reproduction and Development*.

[3] Tilmann C., Capel B. (1999). Mesonephric cell migration induces testis cord formation and Sertoli cell differentiation in the mammalian gonad. *Development*.

[4] Hua J., Sidhu K. (2008). Recent advances in the derivation of germ cells from the embryonic stem cells. *Stem Cells and Development*.

[5] Ata B., Seyhan A., Seli E. (2019). Diminished ovarian reserve versus ovarian aging: overlaps and differences. *Current Opinion in Obstetrics & Gynecology*.

[6] Regan S. L. P., Knight P. G., Yovich J. L., Leung Y., Arfuso F., Dharmarajan A. (2018). Involvement of bone morphogenetic proteins (BMP) in the regulation of ovarian function. *Vitamins and Hormones*.

[7] Mahbod E. (2011). Pathogenesis and causes of premature ovarian failure: an update. *International Journal of Fertility & Sterility*.

[8] Mousavi-Jarrrahi S. H., Kasaeian A., Mansori K., Ranjbaran M., Khodadost M., Mosavi-Jarrahi A. (2013). Addressing the younger age at onset in breast cancer patients in Asia: an Age-Period-Cohort analysis of fifty years of quality data from the international agency for research on cancer. *ISRN Oncology*.

[9] Bedoschi G., Navarro P. A., Oktay K. (2016). Chemotherapy-induced damage to ovary: mechanisms and clinical impact. *Future Oncology*.

[10] Santoro N. (2003). Mechanisms of premature ovarian failure. *Annales d'endocrinologie*.

[11] Akogullari D., Uluer E. T., Vatansever H. S. (2018). Investigation of the relation between follicular atresia and granulosa cells in terms of cell death mechanisms in premature ovarian failure model. *Proceedings*.

[12] Chang E. M., Lim E., Yoon S. (2015). Cisplatin induces overactivation of the dormant primordial follicle through PTEN/AKT/FOXO3a pathway which leads to loss of ovarian reserve in mice. *PLoS One*.

[13] Kalich-Philosoph L., Roness H., Carmely A. (2013). Cyclophosphamide triggers follicle activation and “burnout”; AS101 prevents follicle loss and preserves fertility. *Science Translational Medicine*.

[14] Machura P., Grymowicz M., Rudnicka E. (2018). Premature ovarian insufficiency - hormone replacement therapy and management of long-term consequences. *Menopausal Review*.

[15] Chae-Kim J. J., Gavrilova-Jordan L. (2019). Premature ovarian insufficiency: procreative management and preventive strategies. *Biomedicines*.

[16] Mushahary D., Spittler A., Kasper C., Weber V., Charwat V. (2018). Isolation, cultivation, and characterization of human mesenchymal stem cells. *Cytometry Part A*.

[17] Kisiel A. H., McDuffee L. A., Masaoud E., Bailey T. R., Esparza Gonzalez B. P., Nino-Fong R. (2012). Isolation, characterization, and in vitro proliferation of canine mesenchymal stem cells derived from bone marrow, adipose tissue, muscle, and periosteum. *American Journal of Veterinary Research*.

[18] Park K. (2018). Functional recovery in spinal cord injury using mesenchymal stem cells. *Journal of Controlled Release*.

[19] Chehelcheraghi F., Chien S., Bayat M. (2019). Mesenchymal stem cells improve survival in ischemic diabetic random skin flap via increased angiogenesis and VEGF expression. *Journal of Cellular Biochemistry*.

[20] Fu X., He Y., Wang X. (2017). Overexpression of miR-21 in stem cells improves ovarian structure and function in rats with chemotherapy-induced ovarian damage by targeting PDCD4 and PTEN to inhibit granulosa cell apoptosis. *Stem Cell Research & Therapy*.

[21] Liu T., Huang Y., Zhang J. (2014). Transplantation of human menstrual blood stem cells to treat premature ovarian failure in mouse model. *Stem Cells and Development*.

[22] Ling L., Feng X., Wei T. (2017). Effects of low-intensity pulsed ultrasound (LIPUS) -pretreated human amnion-derived mesenchymal stem cell (hAD-MSC) transplantation on primary ovarian insufficiency in rats. *Stem Cell Research & Therapy*.

[23] Lee H. J., Selesniemi K., Niikura Y. (2007). Bone marrow transplantation generates immature oocytes and rescues long-term fertility in a preclinical mouse model of chemotherapy-induced premature ovarian failure. *Journal of Clinical Oncology*.

[24] Zhang H., Luo Q., Lu X. (2018). Effects of hPMSCs on granulosa cell apoptosis and AMH expression and their role in the restoration of ovary function in premature ovarian failure mice. *Stem Cell Research & Therapy*.

[25] Yin N., Wang Y., Lu X. (2018). hPMSC transplantation restoring ovarian function in premature ovarian failure mice is associated with change of Th17 /Tc17 and Th17 /Treg cell ratios through the PI3K/Akt signal pathway. *Stem Cell Research & Therapy*.

[26] Ding L., Yan G., Wang B. (2018). Transplantation of UC-MSCs on collagen scaffold activates follicles in dormant ovaries of POF patients with long history of infertility. *Science China Life Sciences*.

[27] Zhao G., Cao Y., Zhu X. (2017). Transplantation of collagen scaffold with autologous bone marrow mononuclear cells promotes functional endometrium reconstruction via downregulating *Δ*Np63 expression in Asherman’s syndrome. *Science China Life Sciences*.

[28] Goldstein R. H., Reagan M. R., Anderson K., Kaplan D. L., Rosenblatt M. (2010). Human bone marrow-derived MSCs can home to orthotopic breast cancer tumors and promote bone metastasis. *Cancer Research*.

[29] Rezaee F., Gibson L. F., Piktel D., Othumpangat S., Piedimonte G. (2011). Respiratory syncytial virus infection in human bone marrow stromal cells. *American Journal of Respiratory Cell and Molecular Biology*.

[30] Rebelatto C. K., Aguiar A. M., Moretão M. P. (2008). Dissimilar differentiation of mesenchymal stem cells from bone marrow, umbilical cord blood, and adipose tissue. *Experimental Biology and Medicine*.

[31] Li J., Mao Q. X., He J. J., She H. Q., Zhang Z., Yin C. Y. (2017). Human umbilical cord mesenchymal stem cells improve the reserve function of perimenopausal ovary via a paracrine mechanism. *Stem Cell Research & Therapy*.

[32] Hessvik N. P., Llorente A. (2018). Current knowledge on exosome biogenesis and release. *Cellular and Molecular Life Sciences*.

[33] Branscome H., Paul S., Khatkar P. (2020). Stem cell extracellular vesicles and their potential to contribute to the repair of damaged CNS cells. *Journal of Neuroimmune Pharmacology*.

[34] Marbán E. (2018). The secret life of exosomes: what bees can teach us about next-generation therapeutics. *Journal of the American College of Cardiology*.

[35] Tkach M., Théry C. (2016). Communication by extracellular vesicles: where we are and where we need to go. *Cell*.

[36] Sun L., Li D., Song K. (2017). Exosomes derived from human umbilical cord mesenchymal stem cells protect against cisplatin-induced ovarian granulosa cell stress and apoptosis _in vitro_. *Scientific Reports*.

[37] Théry C., Zitvogel L., Amigorena S. (2002). Exosomes: composition, biogenesis and function. *Nature Reviews Immunology*.

[38] Raposo G., Stoorvogel W. (2013). Extracellular vesicles: exosomes, microvesicles, and friends. *The Journal of Cell Biology*.

[39] Record M. (2018). Introduction to the thematic review series on extracellular vesicles: a focus on the role of lipids. *Journal of Lipid Research*.

[40] Pathan M., Fonseka P., Chitti S. V. (2019). Vesiclepedia 2019: a compendium of RNA, proteins, lipids and metabolites in extracellular vesicles. *Nucleic Acids Research*.

[41] Lindenbergh M. F. S., Stoorvogel W. (2018). Antigen presentation by extracellular vesicles from professional antigen-presenting cells. *Annual Review of Immunology*.

[42] Lai R. C., Arslan F., Lee M. M. (2010). Exosome secreted by MSC reduces myocardial ischemia/reperfusion injury. *Stem Cell Research*.

[43] Ebrahim N., Mostafa O., El Dosoky R. E. (2018). Human mesenchymal stem cell-derived extracellular vesicles/estrogen combined therapy safely ameliorates experimentally induced intrauterine adhesions in a female rat model. *Stem Cell Research & Therapy*.

[44] Zhang H., Liu K. (2015). Cellular and molecular regulation of the activation of mammalian primordial follicles: somatic cells initiate follicle activation in adulthood. *Human Reproduction Update*.

[45] Adhikari D., Liu K. (2009). Molecular mechanisms underlying the activation of mammalian primordial follicles. *Endocrine Reviews*.

[46] Wang H. B., Xie H. R., Xia G. L. (2002). Inhibin and activin in the mammal-ian ovary. *Journal of Agriculture Biotechnology*.

[47] Manabe N., Goto Y., Matsuda-Minehata F. (2004). Regulation mechanism of selective atresia in porcine follicles: regulation of granulosa cell apoptosis during atresia. *The Journal of Reproduction and Development*.

[48] Johnson A. L., Langer J. S., Bridgham J. T. (2002). Survivin as a cell cycle-related and antiapoptotic protein in granulosa cells. *Endocrinology*.

[49] Mohamed M. S., Bishr M. K., Almutairi F. M., Ali A. G. (2017). Inhibitors of apoptosis: clinical implications in cancer. *Apoptosis*.

[50] Choudhary G. S., Al-harbi S., Almasan A. (2015). Caspase-3 activation is a critical determinant of genotoxic stress-induced apoptosis. *Apoptosis and Cancer*.

[51] Friedenstein A. J., Piatetzky-Shapiro I. I., Petrakova K. V. (1967). Osteogenesis in transplants of bone marrow cells. *Journal of Embryology and Experimental Morphology*.

[52] He Y., Chen D., Yang L., Hou Q., Ma H., Xu X. (2018). The therapeutic potential of bone marrow mesenchymal stem cells in premature ovarian failure. *Stem Cell Research & Therapy*.

[53] Liu J., Zhang H., Zhang Y. (2014). Homing and restorative effects of bone marrow-derived mesenchymal stem cells on cisplatin injured ovaries in rats. *Molecules and Cells*.

[54] Yin N., Zhao W., Luo Q., Yuan W., Luan X., Zhang H. (2017). Restoring ovarian function with human placenta-derived mesenchymal stem cells in autoimmune-induced premature ovarian failure mice mediated by Treg cells and associated cytokines. *Reproductive Sciences*.

[55] Chen C. P. (2014). Placental villous mesenchymal cells trigger trophoblast invasion. *Cell Adhesion & Migration*.

[56] Sun M., Wang S., Li Y. (2013). Adipose-derived stem cells improved mouse ovary function after chemotherapy-induced ovary failure. *Stem Cell Research & Therapy*.

[57] Maas S. L. N., Breakefield X. O., Weaver A. M. (2017). Extracellular vesicles: unique intercellular delivery vehicles. *Trends in Cell Biology*.

[58] Desrochers L. M., Bordeleau F., Reinhart-King C. A., Cerione R. A., Antonyak M. A. (2016). Microvesicles provide a mechanism for intercellular communication by embryonic stem cells during embryo implantation. *Nature Communications*.

[59] Corrigan L., Redhai S., Leiblich A. (2014). BMP-regulated exosomes from Drosophila male reproductive glands reprogram female behavior. *The Journal of Cell Biology*.

[60] Wang J., Silva M., Haas L. A. (2014). C-elegans ciliated sensory neurons release extracellular vesicles that function in animal communication. *Current Biology*.

[61] Kaiser J. (2016). Malignant messengers. *Science*.

[62] Belov L., Matic K. J., Hallal S., Best O. G., Mulligan S. P., Christopherson R. I. (2016). Extensive surface protein profiles of extracellular vesicles from cancer cells may provide diagnostic signatures from blood samples. *Journal of Extracellular Vesicles*.

[63] Yim N., Ryu S. W., Choi K. (2016). Exosome engineering for efficient intracellular delivery of soluble proteins using optically reversible protein–protein interaction module. *Nature Communications*.

[64] Qian X., Xu C., Fang S. (2016). Exosomal microRNAs derived from umbilical mesenchymal stem cells inhibit hepatitis C virus infection. *Stem Cells Translational Medicine*.

[65] Balusu S., Van Wonterghem E., De Rycke R. (2016). Identification of a novel mechanism of blood–brain communication during peripheral inflammation via choroid plexus-derived extracellular vesicles. *EMBO Molecular Medicine*.

[66] Boilard E. (2018). Extracellular vesicles and their content in bioactive lipid mediators: more than a sack of microRNA. *Journal of Lipid Research*.

